# Fluid challenge with shock

**DOI:** 10.1186/cc13344

**Published:** 2014-03-17

**Authors:** V Inal, O Mert

**Affiliations:** 1 Trakya University Medical Faculty, Edirne, Turkey

## Introduction

The latest sepsis guideline has emphasized early resuscitative fluid management [1]. Early goal-directed therapy (EGDT) has been shown to improve 28-day mortality in recent studies [2],[3]. This strategy was based on improving tissue perfusion and oxygenation in spite of other supportive and therapeutic measures. Technically and historically, central venous pressure (CVP) measurement is one of the most dependent methods to estimate fluid responsiveness and intravascular volume status on resuscitation. The Surviving Sepsis Campaign (SSC) guidelines recommended goal levels of CVP 8 to 12 mmHg in order to obtain appropriate tissue perfusion [1]. In this study, we objected to re-evaluate effectiveness of a fluid resuscitation strategy in sepsis, comparing the effect of patients' daily fluid balances (DFB) and CVP on patients' survival.

## Methods

Patient records (APACHE II, length of stay (LOS), CVP, DFB, vasopressor and ventilator needs) were retrospectively collaborated, and a randomly-assigned 100 (63 men and 37 women, age 64.2 ± 15.5 years) were statistically analyzed for survival function.

## Results

The mean APACHE II score was 23.6 ± 7.7, LOS was 9.7 ± 10.0 days, intubated period was 6.4 ± 8.6 days, vasopressor period was 4.7 ± 5.5 days, CVP was 10.5 ± 5.5 mmHg, DFB was 1,147.9 ± 1,157.6 ml, and 42 survived. Kaplan-Meier survival and COX regression analysis showed that CVP levels of 6 to 9 mmHg and DFB +800 to +900 ml, but not above, significantly predicted survival, and also shorter LOS, intubated days and lower vasopressor needs with earlier discharge possibility. On the other hand, over-increased DFB and CVP levels strictly correlated with longer LOS and higher mortality rates, and the first 24-hour mean fluid balance alone was surprisingly not predictive.

## Conclusion

Fluid resuscitation therapy is a double-edge-sword. (1) Despite lower volumes, higher volumes also increase mortality. (2) Overall, DFB seemed more important than the first 24-hour DFB.
